# A Clinical Lecture

**Published:** 1888-06

**Authors:** D. A. K. Steele

**Affiliations:** Professor of Orthopedic Surgery in the College of Physicians and Surgeons, Chicago, Illinois; Attending Surgeon to the Cook County Hospital, etc.


### Clinic Reports.

### CLINICAL LECTURE.

By D. A. K. STEELE, M. D.,
Professor of Orthopedic Surgery in the College of Physicians and Surgeons, Chicago, Illinois; Attending Surgeon to the Cook County Hospital, etc.

### TUBERCULOUS ABSCESS IMPLICATING THE ELBOW-JOINT.

*Gentlemen*—Here is a lady who has some trouble with her right elbow, which commenced, she says, about five years ago. On examination I find a sinus leading down to the elbow-joint, discharging a thin, shreddy pus. I find over the internal condyle, particularly, a well-defined, large, oval swelling surrounding the sinus, which gives a rather indistinct sense of fluctuation. There are also evidences of thickening about the joint, peri-articular inflammation and resultant infiltration. You notice also that she has a swelling on the left side of the neck. The swelling implicating the elbow-joint occurred first, then that of the neck.

One of the clinical assistants informs me that she has had scrofulous trouble since she was a child.

Now the diagnosis in a case of this kind becomes comparatively easy. From the number of cases, with a somewhat similar history, that have been presented to the class during the winter and spring, you will all undoubtedly make one diagnosis. And what is it? *Tuberculosis.* Why is it tuberculosis? Because of its history, its insidious developments, of its chronic character, the nature of the discharge and the microscope revealing the presence of Koch's bacillus.

The patient says she is losing flesh, and that her sleep is disturbed on account of the pain which the arm gives her.

It will be necessary to open this abscess near the elbow-joint freely and scrape out, if possible, all the tuberculous matter and diseased tissue. We will find a mass of pale, flabby granulations filling the abscess cavity. These and all infected tissue must be removed.

The patient has just incidentally informed me that she is pregnant. In the administration of an anæsthetic to a pregnant woman there is always a certain amount of danger to be guarded against: *First*, there are the toxic effects of the anæsthetic upon the mother's blood and incidentally to the child; *second*, the unconscious struggles of the mother under anæsthesia, causing traumatism over the uterus, which may induce a miscarriage; *third*, from the administration of ether, the persistent vomiting that is sometimes brought about may be sufficient to induce premature labor. For these reasons, where there is no special urgency, if an operation can be postponed, it is perhaps well to do so. However, it is my experience that the accidents occurring from the administration of an anæsthetic to a pregnant woman are over-estimated. I have in quite a number of instances given anæsthetics under such circumstances, and, so far as I can remember, do not know of a single untoward circumstance or accident occurring. I know of a number of cases where grave operations have been done, in which pregnancy co-existed, and from the nature or gravity of the operation we would anticipate a premature expulsion of a viable fœtus. I remember instances where an ovarian tumor—pregnancy co-existing—was removed, and the woman was delivered at full term of a healthy child, pregnancy having advanced some three or four months at the time of the operation. Other operations of less gravity are recorded, where the patient experienced no unfavorable symptoms whatever, so that in this case, with ordinary care, I would not advise a postponement of the operation. I would not hesitate to advise this patient to submit to the necessary surgical procedure for the removal of as much of the diseased joint as may be deemed expedient.

In previous clinical lectures I called your attention to the fact that when there is disease of the articular surfaces requiring removal of bone, where the disease had extended to the articular ends of the bone, or where the primary nidus was in the epiphises, that partial excision of the elbow-joint does not give good results, that nearly all such cases required subsequent operations. There are undoubtedly some exceptions to this rule, but the general rule I have followed is that, if an elbow-joint requires excision, it requires a total removal of all articular surfaces. We cannot remove the external condyle and leave the internal condyle intact, even if, at the time of the operation, it is apparently not diseased.

I think in this case the entire capsule of the joint should be removed, the articular surfaces and all infected tissue also, so far as it is possible to do so, and that all remaining suspicious-looking tissue should be thoroughly seared over with Paquelin's cautery. At present, however, I do not advise excision of the joint on account of the co-existing pregnancy. We will simply make a free incision, giving exit to the pus, and thoroughly curette the tuberculous tissue, packing the bottom of the wound subsequently with iodoform gauze. If we find the bones are diseased to the extent that I think they are, we will not make an excision of the joint to-day, but postpone it until four or five months after the termination of pregnancy, believing that by so doing I am following my invariable rule of consulting the best interests of this patient.

### TUBERCULOUS ABSCESS OF THE TARSUS.

The next case we have to present to you is also, in all probability, one of tuberculous abscess. The history, as far as can be ascertained, is something like this: Patient is 40 or 45 years of age. Two or three years ago, while out in a yard, some boys playing ball threw the ball in his direction, and he seeing it coming towards him tried to stop it by throwing his right foot up, receiving the ball on the outer surface of the instep or tarsus. He paid little attention to it at first, but two or three weeks later he noticed he was lame. The soreness, lameness and swelling gradually increased. He applied, we are informed, various liniments and local applications to the contusion of the foot, but, notwithstanding that, it has gradually grown worse. At first glance it would seem to a great many of you as though he were suffering from a Pott's fracture. You notice there is eversion of the foot, a prominence of the internal malleolus; there is also softness of the tarsus. He gives no history of injury preceding the blow from the ball. Let us examine this softness, resembling in some degree the extravasation of blood in a recent case beneath the treatment that comes from rupture of minute capillary vessels in the connective tissue. We have here an indistinct, soft, doughy feel, slightly fluctuant, moderately painful, and careful examination shows that we have by palpation an impulse which can be transmitted to the outer side of the foot. With this history of a man at this time of life, a slight injury at first gradually increasing in its severity, lameness and swelling coming on, and suppuration undoubtedly now present, it can mean but one thing, probably, and that is *abscess of the tarsus*, possibly not communicating with the ankle-joint, possibly only implicating one, two or more of the seven little bones that make up the arch of the foot.

I propose to make an exploratory incision into the tarsus over the point of greatest prominence of swelling. If we have to deal with a tuberculous abscess—and from external appearances we have—we will give free exit to the shreddy pus and tuberculous granulations that line all abscess cavities of this sort—those soft, flabby, easily broken down granulations to which I have called your attention in my lectures from time to time—then with a sharp spoon curette clean out the abscess-cavity. If we find erosion of cartilage, or that we have soft or carious bone, tubercular ulceration or destruction of some of the tarsal bones, the spongy, infected portion of them will be removed—in short, all the parts that are diseased, paying but little attention in the operation of following the anatomical outlines of bone. We will go for diseased tissue wherever it can be found without regard to the direction in which it extends, even though we may have to open up the ankle-joint. It is a good rule to remove all the diseased tissue. If, after clearing out the abscess-cavity, we find what I have described, we will pass a drainage tube from side to side so as to afford free drainage. We can cut and lacerate the lateral aspects of the foot without interfering with the powers of locomotion.

